# A new bed side test for inflammatory arthritis

**DOI:** 10.2478/rir-2023-0016

**Published:** 2023-07-22

**Authors:** Saba Samreen, Babur Salim, Haris Gul, Shahida Parveen

**Affiliations:** Rheumatolgy Department, Foundation University School of Health Sciences, Rawalpindi, Punjab 44000, Pakistan

Inflammatory arthritis is characterized by pain, swelling and early morning stiffness in the affected joints. This group of arthritis have the propensity to lead to erosions and joint damage.^[1]^ The resulting joint damage and functional disability not only leads to morbidity but adds to the burden on health care infrastructure. This is an area of particular concern especially in developing countries where the health care facilities and budget is scarce.^[2]^ There are several tools to detect synovitis. Out of these magnetic resonance imaging (MRI) has a fair sensitivity and specificity but cost hampers it is regular use in daily practice especially in developing countries. Similarly musculoskeletal ultrasound also picks up synovitis early in the disease with acceptable sensitivity and specificity^[3]^ but it is not yet commonly available in all setups. Therefore, we need to maximally rely on examination skills especially in developing countries. Timely diagnosis of synovitis and prompt initiation of treatment^[4]^ is the key to better patient related outcomes.

**Figure 1:**
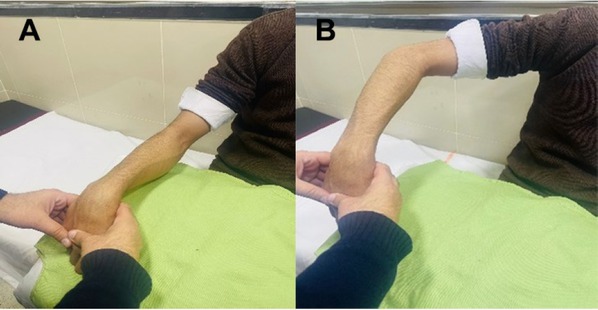
Demonstration the new bedside test: Hyperflexion of the wrist joint in patients with synovitis (A) led to elevation of elbow and abduction of the shoulder joint (B).

Babur sign is one such simple bedside test that can pick synovitis in patients with active arthritis. It can easily be demonstrated when hyperflexion of the wrist performed by the physician results in shoulder abduction along with elevation of the elbow in patient at once as shown in Figure 1. This sign has earlier been validated in patients with rheumatoid arthritis and had a sensitivity and specificity of 87.84% and 78.13% respectively.^[4]^

We demonstrated the sensitivity and specificity of this new bed side test in patients presenting with inflammatory arthritis involving wrist joint. We utilized musculoskeletal ultrasound as a gold standard. This study was conducted at rheumatology outpatient clinics at Fauji Foundation Hospital, Rawalpindi from June 2022 to December 2022 after the approval from ethical committee. Written informed consent was taken from all the patients. A total of 240 patients were included in the study. Patients aged (12-60 years old) were included in the study. These patients had inflammatory arthritis involving wrist joint along with history of morning stiffness lasting greater than an hour. Patients with recent trauma, prior fracture, ankylosed wrist joint, known osteoporosis, soft tissue issues (tendinopathies, ganglion *etc*.) were excluded from the study.

Each patient was examined for synovitis strategically by:

1. examining for joint line tenderness following standard joint examination;
2. musculoskeletal ultrasound examination to pick synovitis;
3. Babur sign performed by two independent observers. These observers were blinded to the results of the first two examinations.

These results were analyzed using SPSS V19.0. Sensitivity and specificity of Babur’s sign for diagnosing synovitis were calculated for inflammatory arthritis.

A total of 240 patients were screened. Out of these 230 (95.8%) patients were female. The mean age of patients (years ± SD) was 35 ± 36.12. Out of these, 140 (58.3%) patients had rheumatoid arthritis, 40 (16.6%) had juvenile idiopathic arthritis, 33 (13.7%) had psoriatic arthritis and 27 (11.2%) had systemic lupus erythematosus (SLE). The sensitivity and specificity of Babur sign for each of these arthritis is as shown in Table 1.

**Table 1:** Sensitivity and specificity of Babur sign in inflammatory arthritis using ultrasound as a gold standard

**Inflammatory arthritis**	**Total patients (N)**	**True positive (TP)**	**True negative (TN)**	**False positive (FP)**	**False negative (FN)**	**Sensitivity (SN)**	**Specificity (SP)**
Rheumatoid arthritis (RA)	140	77	43	10	10	88.5%	81.1%
Psoriatic arthritis (PsA)	33	20	8	3	2	87.0%	80.0%
Juvenile idiopathic arthritis (JIA)	40	14	18	3	5	82.4%	78.3%
Systemic lupus erythematosus (SLE)	27	9	10	3	5	75.0%	66.7%
Total	240	120	79	19	22	84.5%	80.6%

This new bed side test has proven valuable to detect synovitis in clinical practice. The initial results demonstrated its remarkable ability to detect synovitis in patients with rheumatoid arthritis (RA).^[5]^ As seen in these results, comparable sensitivity and specificity can be replicated when applied to other inflammatory arthritis like psoriatic arthritis. Though many studies show imaging techniques like ultrasound and MRI scans to be superior to bed side examination techniques for detecting synovitis.^[6]^ While cost hampers the regular use of MRI to detect synovitis, ultrasound also has its pitfalls such as need of experience and expertise by the physician performing it and subjectivity of the repetitive test. ^[7,8]^ Indeed, we need to improve the clinical expertise for detecting synovitis in patients with inflammatory arthritis and Babur sign is one such text which has proven itself worthy time and again.
